# Switching Signals in the Brain

**DOI:** 10.1371/journal.pbio.0030210

**Published:** 2005-05-03

The brain is arguably the most complex computing machine on the planet, with billions of individual neurons that process the input they receive from sensors in the body and from other neurons. The composition of channels that regulate electrical conductance across the neuronal membrane is a key determinant of the exact pattern of a neuron's response to the stimuli it receives. Bursting—brief epochs of rapid firing—is one such pattern, while single spiking, with longer intervals between firing, is another.

In a deep-seated part of the cerebral cortex, the hippocampus receives input from and sends output to widespread regions of the brain; information processing in the hippocampus is thought to underlie aspects of memory. The output of the hippocampus is handled by a subregion, the subiculum, that exhibits response modes that correspond to either bursting or single-spike response patterns. In this issue, Don Cooper, Sungkwon Chung, and Nelson Spruston show that the bursting–spiking switch is controlled by a new and surprisingly simple mechanism, prolonged inactivation (shutting off) of channels that conduct sodium ions through the membrane.

Working with rats, the authors measured activity from bursting subicular neurons in vivo, and observed frequent transitions from bursting to single spiking. Bursting tended to occur after long silent periods, while single spiking predominated after short intervals. To determine what drove this change from bursting to single spiking, they examined electric activity in brain slices. The switch was strongest when neurons were stimulated at frequencies between 1 and 10 Hertz, which suggested an inactivation and recovery process, perhaps mediated by prolonged inactivation of a small number of sodium channels. To test this hypothesis, the authors applied a very low concentration of tetrodotoxin, a neurotoxin from puffer fish that specifically blocks sodium channels. Indeed, blocking 16% of the channels with the toxin induced a switch from bursting to single spiking even at very low frequency stimulation, when subicular neurons normally maintain their bursting pattern.

Output mode switching by the sustained inactivation of sodium channels is a novel mechanism for controlling the dynamics of neural networks. While the functional significance of the switch remains unexplored, the authors point out that bursting is known to effectively activate target structures. It may be that switching from bursting to single spiking sustains activation of the target once it has been “woken up” by bursting. Conversely, transitions from a powerful burst output to less powerful single-spike mode may serve to initially activate target structures but then allow other inputs to govern the target output. Given the importance of the hippocampus in processing memory and emotion, and its involvement in schizophrenia, epilepsy, and other disorders, these new insights into the regulation of its output may lead to a better understanding of numerous fundamental higher brain processes.[Fig pbio-0030210-g001]

**Figure pbio-0030210-g001:**
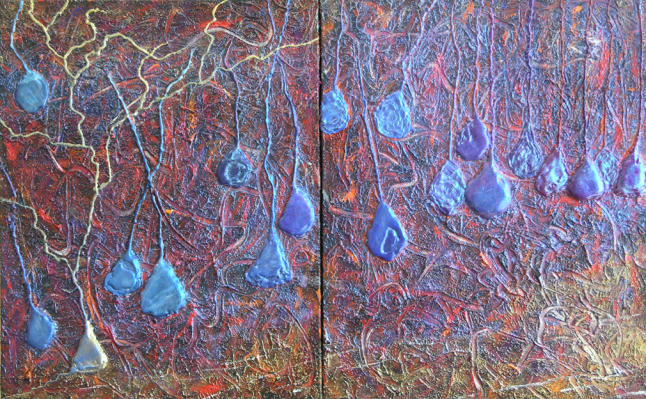
An acrylic painting by Don Cooper and Leah Leverich shows the transition zone between the densely packed pyramidal neurons in the CA1 region (right) and the spread-out pyramidal neurons within the subiculum (left)

